# Immunophenotyping characteristics and outcome of COVID‐19 patients: peripheral blood CD8+T cell as a prognostic biomarker for patients with Nirmatrelvir

**DOI:** 10.3389/fimmu.2023.1227905

**Published:** 2023-09-20

**Authors:** Yuming Sun, Yating Dian, Qian Gao, Guangtong Deng

**Affiliations:** ^1^ Department of Dermatology, Xiangya Hospital, Central South University, Changsha, China; ^2^ National Engineering Research Centre of Personalized Diagnostic and Therapeutic Technology, Changsha, China; ^3^ Furong Laboratory, Changsha, Hunan, China; ^4^ Hunan Key Laboratory of Skin Cancer and Psoriasis, Hunan Engineering Research Centre of Skin Health and Disease, Xiangya Hospital, Central South University, Changsha, China; ^5^ National Clinical Research Centre for Geriatric Disorders, Xiangya Hospital, Changsha, China; ^6^ Department of Plastic and Cosmetic Surgery, Xiangya Hospital, Central South University, Changsha, China; ^7^ Clinical Laboratory Department, Xiangya Hospital, Central South University, Changsha, China

**Keywords:** COVID-19, lymphocyte, T cell, biomarker, CD8+ T cell

## Abstract

**Background:**

Nirmatrelvir has been authorized for the treatment of both hospitalized and non-hospitalized COVID-19 patients. However, the association between T lymphocyte subsets and the outcome of hospitalized COVID-19 patients treated with oral Nirmatrelvir has not been investigated. The objective of this study was to examine whether lymphocyte subsets could serve as biomarkers to assess the risk of mortality in COVID-19 patients undergoing Nirmatrelvir treatment, with the aim of enhancing medication management for COVID-19 patients.

**Methods:**

We conducted a retrospective cohort study at the Xiangya Hospital of Central South University in China between December 5, 2022 and January 31, 2023. The study reported demographic, clinical, T lymphocyte subsets, and inflammatory cytokine data of COVID-19 patients. We evaluated the associations of T lymphocyte subsets on admission with the composite outcome or death of patients using univariate and multivariable Cox regression analyses with hazards ratios (HRs) and 95% confidence intervals (CIs).

**Results:**

We identified 2118 hospitalized COVID-19 patients during the study period, and conducted a follow-up of up to 38 days. Of these, 131 patients received Nirmatrelvir, with 56 (42.7%) in the composite outcome group, and 75 (57.3%) in the non-composite outcome group. Additionally, 101 (77.1%) patients were discharged, while 30 (22.9%) died. Our results showed a significant decrease in the CD3+, CD4+, and CD8+ T cell counts of patients in the composite outcome group and mortality group compared to the non-composite outcome group and discharged group, respectively. Multivariate Cox regression analysis showed that the significant decrease in CD8+ T cell count in peripheral blood was independently associated with the composite outcome in COVID-19 patients treated with Nirmatrelvir, with an HR of 1.96 (95%CI: 1.01-3.80). The significant decrease in CD4+ and CD8+ T cell counts in peripheral blood increased the hazard of developing mortality, with HRs of 6.48 (95%CI: 1.47-28.63) and 3.75 (95%CI: 1.27-11.11), respectively.

**Conclusion:**

Our study revealed a significant positive correlation between a decrease in CD8+ T cell counts and progression and mortality of hospitalized COVID-19 patients treated with Nirmatrelvir. Lower counts (/μL) of CD8+ T cell (<201) were associated with a higher risk of in-hospital severity and death. Our findings may provide valuable references for physicians in optimizing the use of Nirmatrelvir.

## Introduction

Severe Acute Respiratory Syndrome Coronavirus 2 (SARS‐CoV‐2) is regarded as one of the most critical threats to human health and the global economy. Since its first report in 2019, its impact will continue as the Omicron strain mutates and spreads (1). Although COVID-19 initially invades the respiratory system, neurological manifestations have been reported in most patients, including anosmia, ageusia, headache, fatigue, myalgia, dizziness, and psychiatric disorders (2, 3). However, it is still necessary to understand whether the clinical manifestation of COVID-19 patients will change as Omicron mutates. Current evidence shows that COVID-19 infection induces a rapidly coordinated immune response and subsequent inflammatory cytokine storm, which is associated with disease progression (3–5).

Several studies have confirmed the impaired immune system caused by COVID-19 and a significant reduction of lymphocytes, especially a decrease of B cells, natural killer (NK) cells, CD3+ T cells, CD4+ T cells and CD8+ T cells in peripheral blood examination (3, 6–8). At the beginning of the COVID-19 outbreak, some early studies suggested that T lymphocyte cells and their subsets could predict disease severity and mortality (4, 8, 9). Cantenys‐Molina et al. (10) suggested that decreased CD4+ and CD8+T cell counts and expansion of NK lymphocytes proportion at admission were prognostic factors for COVID-19 patients. Mirsharif et al. (3) included 100 COVID-19 patients compared to 70 healthy controls to identify biomarkers for assigning the risk of mortality, and demonstrated that CD8+ HLA‐DR+ T cells count significantly decreased in severe patients and may be the best biomarker for mortality outcome. However, it is unclear whether T lymphocyte subsets can predict the outcome of COVID-19 patients who are treated with antiviral drugs.

Nirmatrelvir is an oral antiviral drug that can effectively inhibit the SARS-CoV-2 3-chymotrypsin–like cysteine protease enzyme (11). Nirmatrelvir has been authorized for COVID-19 patients in many countries worldwide (12, 13). Current clinical evidence suggests that Nirmatrelvir can reduce the risk of 28-days hospitalization or death of outpatients, while showing a well-curative effect in hospitalized patients (12–14). However, the supply of Nirmatrelvir cannot meet the global COVID-19 patients’ demand, and it needs to be used for the most suitable patients. Therefore, in this retrospective study, we focus on the clinical manifestations and immunophenotyping characteristics of COVID-19 patients in the context of the prevalence of Omicron variants. Additionally, we aimed to investigate the potential use of lymphocyte subsets as biomarkers to evaluate the risk of mortality in COVID-19 patients who were undergoing Nirmatrelvir treatment.

## Materials and methods

### Study design and participants

We performed a retrospective, single-center cohort study at Xiangya hospital from December 5, 2022 to January 31, 2023. The study included hospitalized patients with a positive RT-PCR for SARS-CoV-2 infection who received Nirmatrelvir treatment and underwent peripheral blood T lymphocyte subset testing via flow cytometry. Participants under the age of 18, those who received antiviral agents other than Nirmatrelvir, or those who received non-invasive or invasive respiratory support upon admission were excluded. The Xiangya Hospital Institutional Review Committee (202002024) approved our research, and all participants in the retrospective cohort study remained anonymous with no need for individual informed consent.

### Data collection

We retrieved electronic health records of COVID-19 patients from the inpatient system of Xiangya Hospital, which included demographic characteristics, admission date, time from symptom onset to admission, time from symptom onset to treatment exposure (within or beyond 5 days), pre-existing conditions, prescription and drug dispensing records, laboratory tests, ICU admission, and date of discharge or death. Collected data were recorded consecutively until the planned time point.

### Outcomes

The primary outcome was a composite outcome of disease progression including non-invasive respiratory support, initiation of endotracheal intubation, ICU admission and all-cause death. The secondary outcome was each of these individual disease progression outcomes. Patient outcomes were recorded from the date of admission to the occurrence of outcome events, the discharge date, or the date of death, whichever came first.

### Statistical analysis

Continuous variables were presented as median and interquartile range (IQR) and analyzed using the Mann-Whitney test, as most laboratory data had a skewed distribution. Categorical variables were presented as counts and proportions and analyzed using the Chi-square test or Fisher’s exact test. The univariate Cox regression model was used to estimate a HR with a 95% confidence interval (CI) for each result between the groups. The multivariable Cox regression model was then used to control for the impact of confounding variables, including gender, age, comorbidities, and severity at admission. The reference range for normal T lymphocyte subsets was based on the standard of Xiangya Hospital (15). All statistical analyses were performed with SPSS (version 26.0, IBM), and R (version 4.2.1). The level of significance was two-tailed 0.05 for statistical tests.

## Results

### Demographic and clinical characteristics of patients

**Figure 1:**
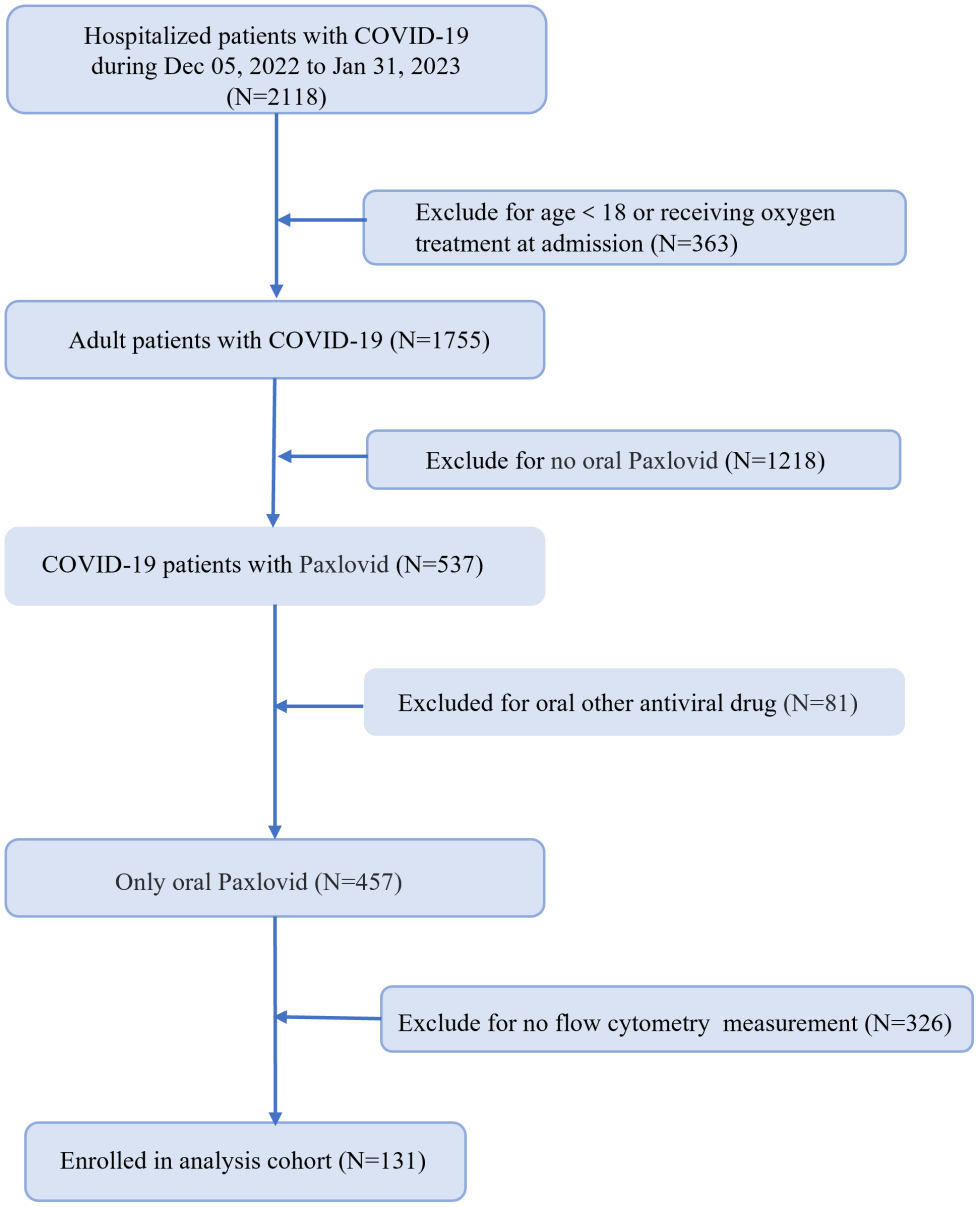
Flowchart of patient recruitment.

Data of 2118 hospitalized patients with confirmed diagnosis of SARS-CoV-2 infection were consecutively collected, and followed up for 38 days. Following the inclusion and exclusion criteria, a total of 131 Nirmatrelvir recipients were enrolled in our cohort (**Figure 1**). **Table 1** showed the demographic and clinical characteristics of patients on admission. Of the enrolled patients, 94 (71.8%) were male and 37 (28.2%) were female with 85 (64.9%) patients aged 65 years or older. Based on disease progression, we divided the COVID-19 patients into two groups - composite outcome (n = 56, 42.7%) and non-composite outcome (n = 75, 57.3%). We found significant differences in gender and age between the two groups (P = 0.031 and P<0.001, respectively). The common clinical symptoms of COVID-19 patients were fever (61.8%), dry cough (86.3%), expectoration (75.6%), poor appetite (48.1%), polypnea (45.8%), fatigue (33.6%), stuffiness (18.3%), myalgia (19.8%), dyspnea (11.5%), and headache (7.6%). On admission, the dry cough, expectoration, and myalgia rates were significantly higher in the non-composite outcome group than in the composite outcome group (P = 0.027, P = 0.003, and P = 0.024, respectively), while the dyspnea rate was lower in the composite outcome group (P = 0.047). Patients with preexisting conditions mainly included hypertension (50.4%), diabetes mellitus (30.5%), coronary disease (26.0%), cancer (13.7%), and chronic obstructive pulmonary disease (9.9%). The rate of patients with hypertension on admission was higher in the composite outcome group than in the no composite outcome group (P = 0.002). However, there was no significant difference in admission severity between the two groups (P = 0.085). Based on medical records, 38 (29.0%) patients received systemic steroid treatment, including 22 (39.3%) in the composite outcome group and 16 (21.3%) in the non-composite outcome group. In addition, 61 (46.6%) patients received antibiotic therapy, and no significant differences were found between the two groups.

**Table 1:** Characteristics of included patients (overall and categorized by disease progression and final outcome).

	Overall (n=131)	No composite outcome (n=75)	Composite outcome (n=56)	P ^1^value	Discharge (n=101)	Died (n=30)	P ^2^value
Gender (M)	94(71.8)	48(64.0)	46(82.1)	**0.031**	68(67.3)	26(86.7)	**0.039**
Age (≥65)	85(64.9)	39(52.0)	46(82.1)	**<0.001**	58(57.4)	27(90.0)	**0.001**
Symptoms, n (%)							
Fever	81(61.8)	46(61.3)	35(62.5)	0.892	64(63.4)	17(56.7)	0.507
Dry cough	113(86.3)	69(92.0)	44(78.6)	**0.027**	91(90.1)	22(73.3)	**0.031**
Expectoration	99(75.6)	65(85.3)	35(62.5)	**0.003**	82(81.2)	17(56.7)	**0.006**
Poor appetite	63(48.1)	37(49.3)	26(46.4)	0.742	49(48.5)	14(46.7)	0.859
Polypnea	60(45.8)	29(38.7)	31(55.4)	0.058	41(40.6)	19(63.3)	**0.028**
Fatigue	44(33.6)	26(34.7)	18(32.1)	0.762	36(35.6)	8(26.7)	0.361
Stuffiness	24(18.3)	13(17.3)	11(19.6)	0.735	18(17.8)	6(20.0)	0.787
Myalgia	26(19.8)	20(26.7)	6(10.7)	**0.024**	24(23.8)	2(6.7)	**0.039**
Headache	10(7.6)	6(8.0)	4(7.1)	1.000	10(9.9)	0 (0)	0.115
Dyspnea	15(11.5)	5(6.7)	10(17.9)	**0.047**	8(7.9)	7(23.3)	**0.043**
Preexisting condition, n (%)							
Hypertension	66(50.4)	29(38.7)	37(66.1)	**0.002**	45(44.6)	21(70.0)	**0.014**
Diabetes mellitus	40(30.5)	19(25.3)	21(37.5)	0.135	30(29.7)	10(33.3)	0.705
Coronary disease	34(26.0)	15(20.0)	19(33.9)	0.072	23(22.8)	11(36.7)	0.156
Cancer	18(13.7)	9(12.0)	9(16.1)	0.503	11(10.9)	7(23.3)	0.127
COPD	13(9.9)	7(9.3)	6(10.7)	0.794	9(8.9)	4(13.3)	0.493
**Severity, n (%)**				0.085			0.189
Mild to moderate	52(39.7)	25(33.3)	27(48.2)		37(36.6)	15(50.0)	
Severe	79(60.3)	50(66.7)	29(51.8)		64(63.4)	15(50.0)	
Oxygen support, n (%)							
Nasal cannula	93(71.0)	67(89.3)	26(46.4)	**<0.001**	84(83.2)	9(30.0)	**<0.001**
Mask oxygen	16(12.2)	0 (0)	16(28.6)	**<0.001**	13(12.9)	3(10.0)	1.000
High-flow oxygen	25(19.1)	0 (0)	25(44.6)	**<0.001**	12(11.9)	13(43.3)	**<0.001**
Invasive mechanical ventilation	27(20.6)	0(0)	27(48.2)	**<0.001**	5(5.0)	22(73.3)	**<0.001**
Medication, n (%)							
Systemic steroid	38(29.0)	16(21.3)	22(39.3)	**0.032**	24(23.8)	14(46.7)	**0.015**
Antibiotics	61(46.6)	32(42.7)	29(51.8)	0.301	40(39.6)	21(70.0)	**0.003**

COPD, chronic obstructive pulmonary disease.

P^1^. Compared between composite outcome and no-composite outcome.

P^2^. Compared between discharge and died.

Bold values indicate a statistical difference in P value.

Patients were categorized into two groups based on their final outcome - discharged (n = 101, 77.1%) or mortality (n = 30, 22.9%). The rate of male and age in the mortality group were significantly higher than in the discharge group (P = 0.039 and P = 0.001, respectively). Furthermore, the common symptom of polypnea and dyspnea was more frequent in the mortality group compared to the discharged group. Of the patients who received oxygen therapy, 93 (71.0%) patients received nasal cannula, 16 (12.2%) patients received mask oxygen, 12 (11.9%) patients received high-flow oxygen, and 5 (5.0%) patients received invasive mechanical ventilation. The rate of receiving high-flow oxygen (43.3%) and invasive mechanical ventilation (73.3%) was significantly higher in the mortality group than in the discharged group. Meanwhile, systemic steroid and antibiotic treatment were more frequent in the mortality group.

### Lymphocyte subpopulations profile and inflammatory cytokines on admission

**Figure 2:**
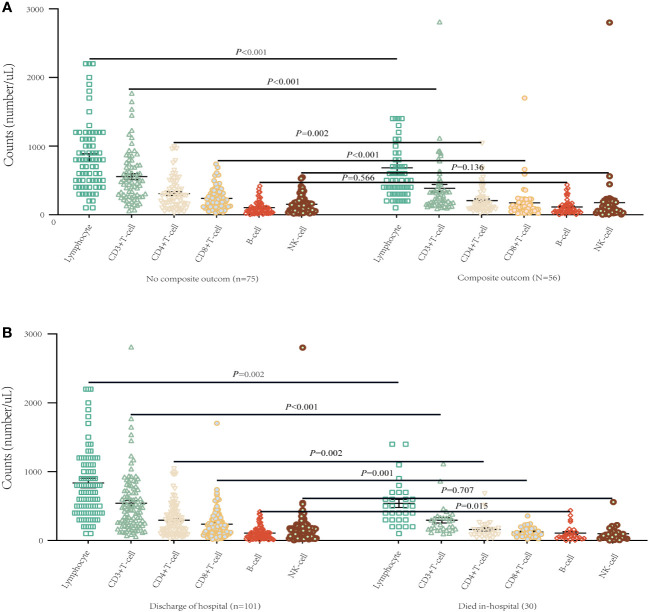
T lymphocyte subsets of different progression of illness and survival conditions in patients on admission with COVID-19. **(A)** Differences of T lymphocyte subsets among composite outcome and no composite outcome-ill patients (mean with SD). **(B)** Differences of T lymphocyte subsets between survivor and non-survivor (mean with SD).

**Table 2** presents detailed characteristics of immune cells. We found that the counts of white blood cells (WBC) and neutrophils were higher in the composite outcome group than in the non-composite outcome group, but the lymphocyte count was lower in the composite outcome group (P < 0.001). Compared with the mortality group, the counts of WBC and neutrophils were lower in the discharged group (P < 0.001), but the lymphocyte count was higher (P = 0.002). Additionally, we found that the levels of T lymphocyte subsets varied according to disease progression and final outcome (**Table 2**). There was a significant decrease in CD3+ T cell, CD4+ T cell, and CD8+ T cell counts in the composite outcome group (**Figure 2A**) and mortality group (**Figure 2B**) compared to the non-composite outcome group and discharged group, respectively. Moreover, we detected the concentration of C-reactive protein (CRP), interleukin-6 (IL-6), and interleukin-10 (IL-10) cytokines produced by T cells in plasma. We found that the concentration of CRP and IL-10 was significantly higher in the composite outcome group (P < 0.01). Notably, the median concentration of CRP, IL-6, and IL-10 in the mortality group was over 2-fold higher than in the discharged group.

**Table 2:** Immunoprofiling of lymphocyte subsets in peripheral blood of COVID‐19 patients.

Immunoprofiling, median (IQR)	Overall (n=131)	No composite outcome (n=75)	Composite outcome (n=56)	P ^1^value	Discharge (n=101)	Mortality (n=30)	P ^2^value
WBC (10^9^/L)	5.70(3.80,8.90)	5.10(3.40,7.50)	7.90(4.80,11.20)	**<0.001**	5.20(3.60,7.60)	9.90(5.20,11.80)	**<0.001**
Neutrophil(10^9^/L)	4.30(2.60, 7.3)	3.60(2.10,5.90)	6.30(3.60,9.60)	**<0.001**	3.80(2.40,6.10)	9.00(3.90,10.90)	**<0.001**
Eosinophil (10^9^/L)	0.01(0,0.05)	0.01(0,0.06)	0(0,0.03)	0.090	0.01(0,0.05)	0(0,0.04)	0.194
Basophil (10^9^/L)	0.010(0.01,0.02)	0.010(0.010,0.020)	0.010(0.003,0.018)	0.462	0.01(0,0.02)	0.01(0.01,0.02)	0.824
Lymphocyte (/uL)	600(400,900)	700(500,1000)	550(400,700)	**<0.001**	700(400,900)	500(300,700)	**0.002**
CD3+ T (/uL)	351.0(207.0, 633.0)	483.0(269.0,727.0)	271.0(166.8,437.8)	**<0.001**	461.0(242.5,725.0)	245.5(163.0,337.3)	**<0.001**
CD4+ T (/uL)	180.0 (85.0, 336.0)	269.0(132.0,408.0)	150.5(67.8,244.3)	**0.002**	237.0(101.5,401.0)	135.0(81.8,188.8)	**0.002**
CD8+T (/uL)	174.0 (89.0, 266.0)	211(114.0,321.0)	108.0(70.3,196.0)	**<0.001**	190.0(104.0,307.5)	98.0(70.8,168.8)	**0.001**
CD4+/CD8+	1.34 (0.80, 1.87)	1.34(0.80,1.79)	1.36(0.81,2.20)	0.633	1.35(0.80,1.90)	1.27(0.84,1.86)	0.844
B cell (/uL)	79.0 (36.5, 146.0)	79.0(28.0,126.0)	78.5(38.0,149.8)	0.566	81.0(36.8,149.0)	62.0(36.0,130.0)	0.707
NK cell (/uL)	114.0 (46.5, 186.5)	133.0(60.0,184.0)	88.5(35.5,190.5)	0.136	142.0(55.3,206.0)	72.0(37.0,119.0)	**0.015**
CRP (mg/L)	56.8(14.6, 107.3)	25.8(6.0,69.0)	76.3(37.6,152.8)	**<0.001**	46.8(12.1,81.3)	97.2(29.9,181.1)	**0.002**
IL-6 (pg/ml)	12.8(3.6,35.9)	11.6(2.6,31.1)	12.9(5.2,45.3)	0.171	11.8(2.8,31.1)	24.3(8.6,98.4)	**0.019**
IL-10(pg/ml)	5.0(2.5,5.9)	5.0(1.8,5.0)	5.7(3.0,13.1)	**0.008**	5.0(1.9,5.0)	10.6(5.6,26.2)	**<0.001**

P^1^. Compared between composite outcome and no-composite outcome.

P^2^. Compared between discharged and died.

Bold values indicate a statistical difference in P value.

### Association of lymphocyte subsets with composite outcome and mortality in COVID‐19 patients

**Figure 3:**
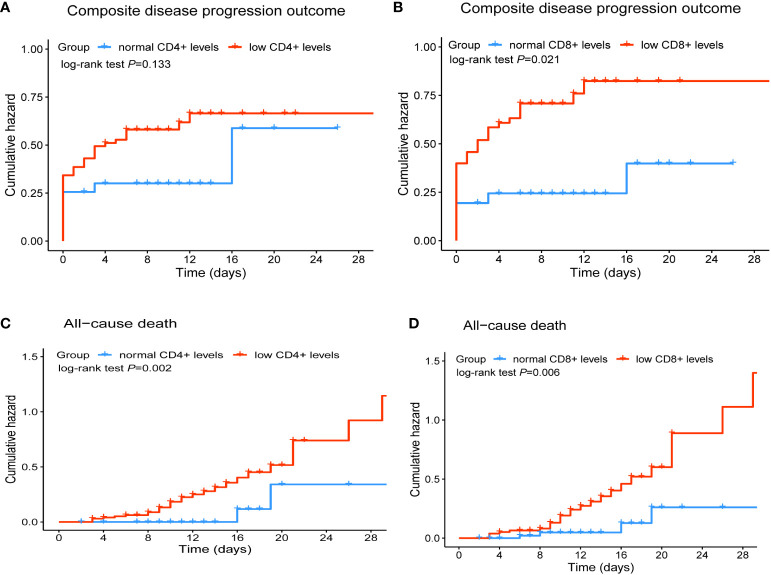
Cumulative incidence of composite disease progression outcome **(A, B)** and all-cause death **(C, D)** of COVID-19 patients for lower CD4+ and CD8+ T cell counts versus normal CD4+and CD8+ T cell counts.

We further explored the association of different lymphocyte subsets with disease progression and final outcome in hospitalized COVID-19 patients treated with Nirmatrelvir. The results of univariate Cox regression were shown in **Table 3**. We found that the risk of in-hospital composite outcome was statistically higher in those who had lower counts of CD8+ T cells (HR = 2.54, 95%CI: 1.34-4.84). Moreover, the lower counts of CD4+ T cells and CD8+ T cells were significantly associated with in-hospital death (HR: 4.61; 95%CI: 1.10-19.38, HR: 3.90; 95%CI: 1.36-11.19, respectively). Furthermore, multivariate Cox regression analysis revealed that a significant decrease in CD8+ T cell count in peripheral blood was independently associated with composite outcome in COVID-19 patients treated with Nirmatrelvir, with an HR of 2.01 (95%CI: 1.03-3.90), adjusted for age, gender, comorbidities, severity at admission and the Nirmatrelvir treatment within or beyond 5 days of symptom onset (**Table 4**). We also found that a significant decrease in CD4+ T cell and CD8+ T cell count in peripheral blood increased the hazard of developing mortality (HR: 6.05; 95%CI: 1.39-26.29, HR: 3.61; 95%CI: 1.22-10.63, respectively), adjusted for age, gender, comorbidities, severity at admission and Nirmatrelvir treatment within or beyond 5 days of symptom onset (**Table 4**). **Figure 3** represents the cumulative risk of developing composite outcome and mortality in COVID-19 patients treated with Nirmatrelvir, whose peripheral blood CD4+ T cell and CD8+ T cell counts decreased significantly at admission.

**Table 3:** Comparison of the distribution of lymphocyte subsets as risk factors of clinical outcome for patients with COVID‐19.

	Composite outcome			Mortality		
	Univariable HR	95% CI	P value	Univariable HR	95% CI	P value
Lymphocyte						
<1.1	1.59	0.78-3.25	0.203	2.59	0.78-8.56	0.118
≥1.1	Ref			Ref		
CD4+T*/*CD8+T/uL						
<0.53	1.43	0.64-3.20	0.390	1.68	0.57-4.93	0.348
0.53-2.31	Ref			Ref		
>2.31	1.41	0.70-2.83	0.333	0.85	0.31-2.32	0.746
CD3+T/uL						
<711	1.89	0.85-4.18	0.118	3.89	0.93-16.35	0.064
711-2353	Ref			Ref		
CD4+T/uL						
<368	1.67	0.82-3.41	0.159	**4.61**	**1.10-19.38**	**0.037**
368-1632	Ref			Ref		
CD8+T/uL						
<201	**2.54**	**1.34-4.84**	**0.004**	**3.90**	**1.36-11.19**	**0.011**
201-931	Ref			Ref		
B-cell/uL						
<74	0.98	0.55-1.76	0.948	1.73	0.80-3.75	0.164
74-534	Ref			Ref		
NK-cell/uL						
<63	1.25	0.68-2.30	0.470	0.90	0.41-1.97	0.789
63-1013	Ref			Ref		

Bold values indicate a statistical difference in P value.

**Table 4:** Multivariable Cox regression for composite outcome and mortality.

	Multivariable HR	95% CI	P value
Composite outcome			
CD8+T/uL (<201)	1.96	1.01-3.80	0.048
All-cause Mortality			
CD4+T/uL (<368)	6.48	1.47-28.63	0.014
CD8+T/uL (<201)	3.75	1.27-11.11	0.017

Multivariable Cox regression adjusted gender, age, comorbidities, and severity at admission.

## Discussion

This retrospective study investigated hospitalized patients during the Omicron variant epidemic. We analyzed the demographic, immunophenotype, and clinical characteristics of 131 COVID-19 patients treated with Nirmatrelvir. Neurological manifestations such as anosmia, ageusia, and psychiatric disorders were not common in enrolled patients. It may also be the widespread of full vaccination, which protects the nervous system from Omicron damage. A recent study shows that children who are not fully vaccinated may develop omicron-related neurological complications (16). In composite-outcome patients, the median counts of lymphocytes decreased, and CD3+ T cell, CD4+ T cell, and CD8+T cell counts were almost decreased to half of median counts of non-composite-outcome patients. Similar results were observed in patients who died in the hospital compared to discharged patients. Meanwhile, inflammatory cytokines were significantly higher in patients with composite outcome and death than in patients with non-composite outcome and discharge, respectively. The median concentrations of CRP, IL-6, and IL-10 in died patients were more than two-fold of that of discharged patients. Previous evidence has shown that lymphocyte subsets significantly reduced, including CD3+T cells, CD4+T cells, and CD8+T cells, B cells, NK cells, and cytokine storms such as CRP are common in COVID-19 patients (8, 10, 17). In this study, we explored the association of peripheral blood T-lymphocyte subsets with the prognosis of COVID-19 patients treated with Nirmatrelvir. We found that lower counts (/μL) of T lymphocyte subsets CD8+T cells (<201) were associated with a higher risk of composite outcome, and lower counts (/μL) of CD4+T cells (<368) and CD8+T cells (<201) were significantly associated with the death outcome of COVID‐19 patients with Nirmatrelvir therapy. Identifying biomarkers that predict the curative effect of Nirmatrelvir may help physicians conduct evidence-based treatments for COVID-19.

The immune response is closely related to the pathogenesis, progression, and prognosis of COVID-19 patients, especially the activation of adaptive immune function (18). CD4+ and CD8+ T cells are the most basic components of adaptive immunity, with various helper and effector functionalities and the ability to kill infected cells respectively (7). Previous studies on CD4+ and CD8+ T cells in COVID-19 patients have mostly focused on the progression and prognosis of the disease from mild to severe. However, there are no reports on CD4+ and CD8+T cells regarding progression and mortality for COVID-19 patients who treated with Nirmatrelvir. One study on 701 COVID-19 patients reported that the counts of CD4+ T cells (≤500) and CD8+ T cells (100) were significantly associated with mortality (10). The earliest study by Du et al. showed a significant decrease in 21 deceased patients compared with 158 survivors, and when CD8+T cells reduced (≤75), the risk of death increased more than five-fold (9). Xu et al. in the evaluation of 187 hospitalized patients with COVID‐19, reported that the counts of total lymphocytes, CD3+ T cells, CD4+ T cells, CD8+ T cells, B cells, and NK cells decreased significantly. And the sensitivity analysis indicated that the count of lymphocytes (<500), CD3+ T cells (<100), CD4+ T cells (<100), CD8+ T cells (<100), and B cells (<50), were risk factors for COVID-19 patients’ death (8). We note that several studies reported the significant decrease in CD4+ T cells, CD8+ T cells of COVID-19 patients with increasing disease severity (17, 19, 20).

Our study also has some limitations. Firstly, we conducted a single-center retrospective study in Hunan province, limiting the generalizability of our findings. Future studies on the association of T lymphocyte subsets with the prognosis of COVID-19 patients in different regions and ethnicities should be carried out. Secondly, the supply of other antiviral drugs was insufficient, and hence we only evaluated the association between T lymphocyte subsets and prognosis in COVID-19 patients treated with Nirmatrelvir. Thirdly, our population of COVID-19 patients was mainly composed of females (71.8%), which may not be sufficient to predict the prognosis of Nirmatrelvir in male patients. Fourth, the absence of data before and after the administration of Nirmatrelvir may limit the ability to fully assess its impact on lymphocyte subsets as biomarkers. Finally, due to the small sample size, we did not perform ROC curve analysis to confirm the warning values of T lymphocytes. To our best knowledge, we are the first to explore the association of T lymphocyte subsets with the prognosis of COVID-19 patients treated with Nirmatrelvir. Flow cytometry is a quick and convenient method to detect peripheral blood lymphocyte subsets for hospitalized COVID-19 patients, which could help to identify the most suitable patients treated with Nirmatrelvir.

## Conclusion

In summary, our study reveals a significant correlation between decreased CD8+ T cell counts and in-hospital progression and mortality of COVID-19 patients treated with Nirmatrelvir. Specifically, patients with lower CD8+ T cell counts (/μL < 201) exhibited a higher risk of in-hospital severity and death. These findings may provide valuable references for physicians in optimizing the use of Nirmatrelvir.

## Data availability statement

The raw data supporting the conclusions of this article will be made available by the authors, without undue reservation.

## Ethics statement

The Xiangya Hospital Institutional Review Committee (202002024) approved this research, and all participants in the retrospective cohort study remained anonymous with no need for individual informed consent.

## Author contributions

Conception and design: GD and QG. Acquisition of data: YS and YD. Interpretation of data, statistical analysis and manuscript writing: YS and GD. Revision of manuscript and administrative, technical, or material support: GD, QG, YS, and YD. All authors contributed to the article and approved the submitted version.
